# Avoiding Preemptive Extracorporeal Membrane Oxygenation in Near-Occlusive Tracheal Chondrosarcoma: An Awake Airway Strategy

**DOI:** 10.7759/cureus.99457

**Published:** 2025-12-17

**Authors:** Kareem Hassan, Ravi Desai, Andrew Nolasco, Mohammad Andalib, Mark Schlesinger

**Affiliations:** 1 Anesthesiology, Hackensack University Medical Center, Hackensack, USA

**Keywords:** airway obstruction, awake intubation, central airway tumor, cook airway catheter, difficult airway management, microlaryngoscopy tube, tracheal chondrosarcoma, tracheal mass, tracheal stenosis, venovenous ecmo

## Abstract

Primary tracheal chondrosarcoma is an exceptionally rare malignancy. When it presents with near-occlusive airway obstruction, it creates substantial anesthetic challenges, particularly regarding safe airway control and potential reliance on extracorporeal support. In the setting of significant tracheal narrowing, some clinicians consider preemptive venovenous extracorporeal membrane oxygenation (VV-ECMO) because induction of anesthesia may precipitate complete airway obstruction. We report a case of a 57-year-old man with >95% tracheal occlusion from a proximal tracheal chondrosarcoma; tumor vascularity and inferior extension made tracheostomy unsafe and prevented advancement of a standard adult bronchoscope. To avoid the risks associated with preemptive ECMO, spontaneous ventilation was maintained under monitored anesthesia care (MAC) with VV-ECMO on standby. A modified awake fiberoptic intubation technique was performed using video laryngoscopy, a microlaryngoscopy tube, and a Cook airway exchange catheter to secure the airway without precipitating obstruction. After successful intubation, general anesthesia was induced, and rigid bronchoscopic debulking proceeded without complications. This case demonstrates that in select patients with near-total tracheal obstruction, a structured hybrid approach combining preserved spontaneous ventilation, topical anesthesia, and low-profile intubation adjuncts can achieve secure airway control while avoiding the risks of preemptive ECMO. This strategy represents a practical alternative when conventional awake fiberoptic intubation is not feasible, underscoring important considerations for tailoring airway management in rare tracheal pathology.

## Introduction

Chondrosarcoma is a rare cancer of the hyaline cartilage-producing chondrocytes and commonly presents as bone tumors, especially in the pelvis or the femur, with an incidence of approximately 9 per million patients [1]. An even rarer subset constitutes those localized to the head and neck, which are only 12% of primary chondrosarcoma cases [2]. Furthermore, there are fewer than 40 known cases of tracheal chondrosarcomas that have been reported in the literature through 2024 [3]. Consequently, the presentation of a proximal tracheal chondrosarcoma is especially uncommon and provides a unique opportunity to explore the airway management challenges associated with its inherently unique nature.

Tracheal masses predominantly occur via secondary spread and are much less frequently primary tumors, two-thirds of which are squamous cell and adenoid cystic carcinomas [4], with the former having a strong association with tobacco use. In general, tracheal malignancies often have a poor prognosis, with 5- and 10-year survival rates of 5%-15% and 6%-7%, respectively [5]. Clinically, they often present with non-specific symptoms such as cough, dyspnea, and stridor at baseline and can be exacerbated by dynamic processes that induce airway narrowing such as swallowing, physical exertion, sleep, respiratory infections, and, more pertinently, anesthetic sedation. Furthermore, at higher degrees of luminal obstruction, symptoms may worsen in severity and may escalate to resting dyspnea and even periods of apnea, creating a life-threatening medical emergency.

Cross-sectional imaging using contrast-enhanced CT or MRI plays a crucial role in characterizing tracheal masses, as vascularity, degree of obstruction, and anatomical relationships directly influence both surgical planning and anesthetic management. These considerations are important when general anesthesia may precipitate complete airway collapse. Historically, airway management for tracheal tumors has included endotracheal intubation under general anesthesia with the tube positioned above or below the lesion or surgical tracheostomy when anatomically feasible.

Over the past decade, extracorporeal membrane oxygenation (ECMO) has emerged as an adjunct for managing anticipated loss of ventilation in severe central airway obstruction [6]. Contemporary reviews recommend considering preemptive venovenous (VV) ECMO when the residual tracheal diameter is <5 mm or when maintaining ventilation during induction is unsafe [7]. These recommendations are supported by multiple case reports describing successful airway management via ECMO in the setting of marked tracheal narrowing with primary tracheal malignancies as the leading indication [8-11]. Nevertheless, large meta-analytic data show that ECMO is accompanied by a high frequency of major complications, with rates of renal failure exceeding 50%, bleeding, and pneumonia each affecting roughly one-third of patients, and limb ischemia occurring in about 10% [12].

Awake fiberoptic intubation remains a mainstay for securing the airway in high-grade intraluminal stenosis; however, severe tracheal narrowing may prevent passage of a standard adult bronchoscope. Several recent studies have explored hybrid strategies that combine video laryngoscopy with flexible bronchoscopy to improve glottic alignment and optimize first-pass success [13]. Cook airway exchange catheters have also been used to facilitate intubation or serve as conduits for oxygenation, though their limited column strength can impede advancement through tight or distorted anatomy, and exchange failures are documented [14].

Despite a growing interest in combined awake techniques, detailed reports that integrate video laryngoscopy, a microlaryngoscopy tube (MLT), and a Cook exchange catheter while maintaining VV-ECMO strictly on standby, in the setting of near-total intraluminal tracheal obstruction, remain scarce. This case contributes to that emerging body of literature by presenting a structured, device-specific awake strategy for a >95% obstructing primary tracheal chondrosarcoma in which airway control was achieved without preemptive ECMO.

## Case presentation

**Figure 1:**
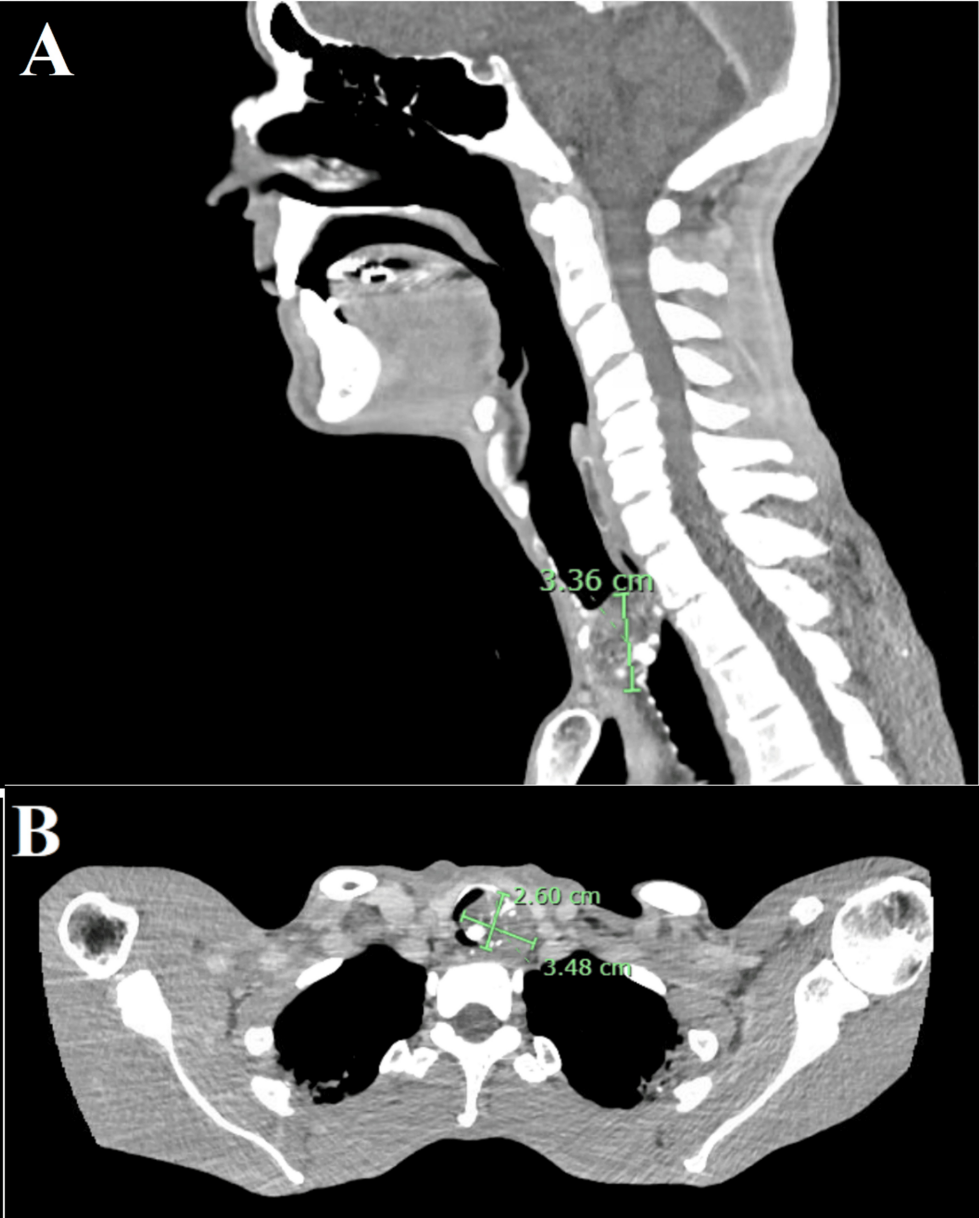
(A) Sagittal and (B) transverse contrast CT of the neck

A 57-year-old man with an American Society of Anesthesiologists (ASA) physical status IV presented initially to an outside ED regarding progressive exertional dyspnea, a 20-lb unintentional weight loss, and an unremitting sinus infection over several months. A chest radiograph and CT of the head revealed mild inflammatory changes in the sphenoid and ethmoid sinuses. The patient was prescribed oral Augmentin and an otolaryngologist referral for further evaluation. At follow-up one week later, a flexible laryngoscopy of the supraglottic and glottic structures revealed no abnormalities but raised concerns for subglottic stenosis due to new onset stridor. Contrast-enhanced CT of the neck and chest revealed a partially calcified tracheal mass 3.48 × 2.6 × 3.36 cm located at the thoracic inlet, causing critical tracheal narrowing and abutment of the esophagus, as shown in Figure 1. Preoperative bedside video bronchoscopy revealed a highly vascular lesion suspicious for malignancy. At the time of admittance, the patient appeared cachectic secondary to increased dyspnea exacerbated by oral dietary intake. The patient had a BMI of 18.1, was alert and cooperative, without cyanosis or digital clubbing. Pre-procedure vitals included BP 108/57 mmHg, pulse 64 beats per minute, temperature 98.7 °F, respiratory rate 47, and SpO₂ 97% on room air. His past medical history was otherwise unremarkable, and he denied previous tobacco use and previous anesthetic complications.

Given the high degree of tracheal narrowing observed (>95%) and risk of absolute occlusion with induction of general anesthesia, the team planned an awake fiberoptic intubation with preservation of spontaneous ventilation and ECMO on standby.

Preoperatively, the cardiac surgery team cannulated the right internal jugular and right femoral veins to allow rapid initiation of venovenous ECMO if required; however, the circuit was not activated and remained on standby. Invasive arterial monitoring was used and large-bore intravenous access was obtained. In the operating room, the patient was positioned supine with slight neck extension to optimize airway visualization. The airway was pretreated with the nebulized topical anesthetic, including cetacaine and 4% lidocaine, to minimize patient discomfort and cough reflex. An antisialagogue, glycopyrrolate 0.4 mg IV, was administered to reduce airway secretions and enhance the effectiveness of topical anesthesia. The thoracic surgeon then continued anesthetizing the upper airway with additional spray-as-you-go aliquots of 2% lidocaine spray through a flexible bronchoscope channel.

A level of moderate sedation was then achieved using incremental doses of midazolam to a total of 4 mg, and remifentanil administered as two boluses of 0.5 mcg/kg followed by a continuous titrated infusion up from 0.02 to 0.15 mcg/kg/min. Reversal agents, flumazenil and naloxone, were prepared and available if required. During this period, the patient remained hemodynamically stable, responsive to light tactile stimulus, and maintained adequate spontaneous ventilation without desaturation.

**Figure 2:**
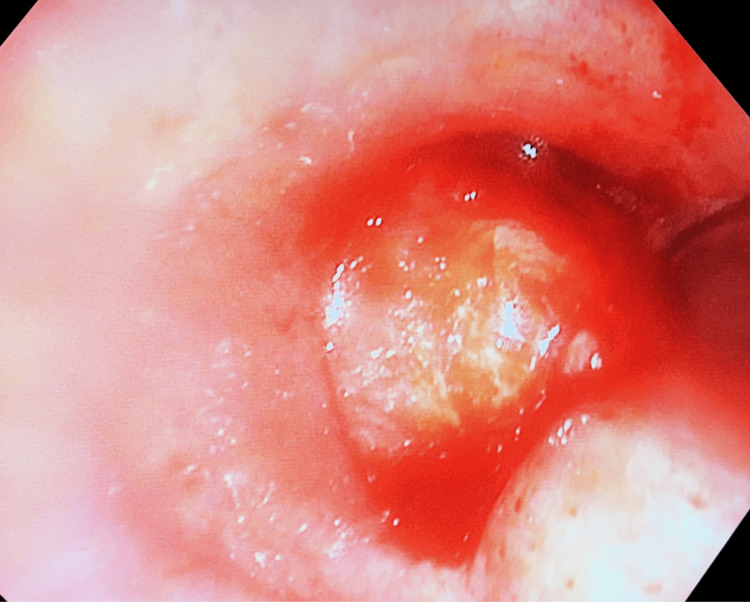
Perioperative image of the tracheal mass

With the appropriate depth of sedation and airway topicalization reached, the cough reflex was sufficiently blunted, and the bronchoscope was successfully advanced through the vocal cords. Intraoperative flexible bronchoscopy examination revealed a large, friable tracheal mass with almost total occlusion of the tracheal lumen (>95%) as shown in Figure 2. Due to the extent of obstruction, the adult bronchoscope was unable to traverse the lesion. Instead, a pediatric bronchoscope was used to circumvent the lesion, allowing direct visualization of the carina and confirming patent airways distal to the lesion. The tracheal lesion was measured intraoperatively to be approximately 3.5 cm in length, extending from the level of the second tracheal ring to approximately 6 cm proximal to the carina.

Definitive airway control was secured using a modified awake fiberoptic intubation that implemented video laryngoscopy and a Cook airway exchange catheter in place of a traditional bronchoscope. The GlideScope video laryngoscope was used to elevate the epiglottis and visualize the glottis. A 6.0-mm MLT with a pre-loaded flexible stylet was advanced through the vocal cords, proximal to the mass, using visual guidance. The flexible stylet was then exchanged for a 14 French Cook catheter, the GlideScope was removed from the mouth, and a flexible bronchoscope was advanced past the vocal cords. Cook catheter size selection was based on its similarity in diameter to the pediatric bronchoscope that was used to traverse past the lesion previously. With careful manipulation and under visual guidance of the flexible bronchoscope, the Cook catheter was traversed distal to the lesion, and the 6.0-mm MLT was carefully railroaded over the Cook catheter and past the lesion. The Cook catheter was then removed, and a pediatric bronchoscope was advanced through the MLT, confirming that the tip lay past the mass and above the level of the carina. The balloon was then inflated, and effective ventilation was verified by continuous capnography and observation of end-tidal CO₂ waveforms. Following this confirmation of a secure airway, general anesthesia was induced, and neuromuscular blockade was administered to facilitate surgical resection of the tracheal mass.

**Figure 3:**
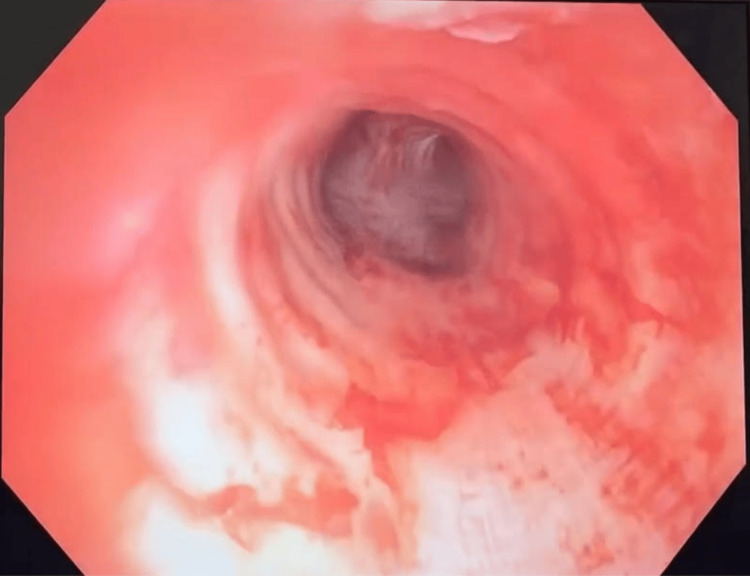
Post-resection perioperative image

The surgical team began with injections of concentrated epinephrine into the mass using a Wang cytology needle to reduce vascularity and minimize intraoperative hemorrhagic potential. A cryobiopsy was then performed for frozen section analysis of the mass. Following this, a 7.5-mm rigid bronchoscope was advanced superior to the mass, and serial mechanical debulking was performed using rigid forceps. Additional tissue specimens were obtained for permanent histopathologic evaluation. Definitive mass removal was performed by placing the bronchoscope at the interface between the tumor and tracheal mucosa and circumferentially coring out the lesion until complete excision was achieved. The post-resection appearance of the tracheal lumen is shown in Figure 3.

Following gross resection and satisfactory hemostasis of the tracheal tissue, a flexible bronchoscope was reintroduced to inspect the distal trachea and mainstem bronchi for fluid and debris removal. With airway caliber restored, the microlaryngoscope was removed, and the patient was reintubated with a standard 8.0-mm endotracheal tube for further inspection and to ensure continued airway patency. The resection field was irrigated with dilute epinephrine, observed for several minutes to confirm hemostasis, and the procedure was concluded uneventfully. The patient was extubated in the OR, monitored in the ICU overnight, and discharged home on postoperative day 1.

## Discussion

Management of this patient was defined by the competing priorities of maintaining airway patency, avoiding preemptive ECMO, and preventing catastrophic loss of ventilation. Tracheostomy was initially considered as a means of securing a distal airway; however, the approach was deemed high-risk due to the tumor’s vascularity, size, and anatomic location. Standard tracheostomies are typically performed between the second and third tracheal rings, safely above the level of the innominate artery [15]. In contrast, the inferior extent of the patient’s mass necessitated a low tracheostomy, placing the operative field dangerously close to the innominate artery. Of note, low tracheostomies below the third tracheal ring are well-documented to significantly elevate the risk of tracheo-innominate fistula formation, a rare but often fatal complication [16]. Furthermore, induction of anesthesia or airway manipulation prior to securing the tracheostomy could have precipitated total airway obstruction and collapse.

High-grade tracheal stenosis presents a unique hazard in which even minimal reductions in diaphragmatic tone or alterations in airway pressure dynamics following induction of general anesthesia can precipitate complete airway obstruction and hypoxemia [17]. For this reason, VV-ECMO is often initiated before airway manipulation to provide oxygenation and CO₂ removal independent of pulmonary ventilation. As a result, many published case reports of near-total tracheal obstruction highlighted the preemptive use of VV-ECMO or cardiopulmonary bypass as a means of mitigating the high risk of airway collapse during induction [8-11]. Building on accumulated case experience, Kim et al. proposed that preemptive ECMO should be strongly considered when the minimal tracheal diameter is <5 mm or when critical oxygen desaturation occurs despite maximal ventilatory support [18], which was later reinforced by the analysis of Maxwell and Forrest [7]. These criteria have since influenced modern airway algorithms.

Despite its growing use, ECMO is associated with a substantial burden of morbidity, with major complications such as renal dysfunction, bleeding, pulmonary infection, and limb ischemia occurring with notable frequency during support [12]. Preemptive ECMO may also unintentionally encourage aggressive airway manipulation under the assumption that extracorporeal support ensures safety. Maneuvers such as forcibly advancing an adult or rigid bronchoscope through a large friable tumor pose significant danger, including hemorrhage and complete obstruction that complicates rescue efforts, even when ECMO is available. Additionally, traumatic endoluminal injury may worsen postoperative edema, impair mucosal healing, and predispose the patient to delayed airway compromise.

**Figure 4:**
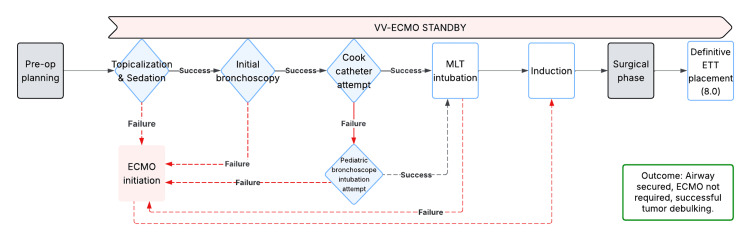
Hybrid awake airway management algorithm with planned VV-ECMO rescue pathways The dashed line denotes the contingency path, and the solid line denotes the success path. VV-ECMO: venovenous extracorporeal membrane oxygenation; MLT: microlaryngoscopy tube; ETT: endotracheal tube

A key distinguishing aspect of this case was the strategic choice to keep VV-ECMO on standby, representing a departure from the conventional practice of preemptive initiation in similar cases. To achieve this, the plan relied on maintaining spontaneous ventilation through monitored anesthesia care and the use of low-profile, atraumatic airway devices. A stepwise outline of the airway management plan and predetermined ECMO escalation pathways is illustrated in Figure 4.

Instead of a traditional awake fiberoptic intubation, which would not be feasible in this setting, the team adopted a hybrid awake technique. Video laryngoscopy provided glottic exposure without occupying the airway lumen, enabling atraumatic placement of an MLT above the lesion. The MLT was selected for its smaller external profile and greater length compared with standard adult endotracheal tubes, improving the likelihood of negotiating the obstruction and ensuring adequate distal positioning once the airway was secured. The Cook airway exchange catheter was then used as a low-profile, pliable guide to traverse the residual lumen under intermittent bronchoscopic confirmation. Unlike a bougie, the Cook catheter preserved the option for oxygen insufflation or jet ventilation if obstruction occurred, providing an additional margin of safety in the event of partial obstruction. The Cook catheter also avoided the potentially traumatic bulk of a bronchoscope used in traditional awake fiberoptic intubation.

By achieving definitive airway control without preemptive ECMO cannulation, this approach avoided ECMO-related complications while preserving an immediate rescue option if obstruction occurred. The strategy underscored that preparedness and restraint are equally critical when managing severe airway pathology. For select patients with similar anatomy and physiology, a carefully coordinated hybrid awake technique may offer a safe alternative to routine preemptive ECMO.

## Conclusions

This case highlights the substantial anesthetic and airway challenges presented by a near-critical tracheal obstruction from an exceptionally rare primary tracheal chondrosarcoma. Severe luminal narrowing, tumor vascularity, and unfavorable anatomy rendered conventional approaches, such as tracheostomy or standard bronchoscopy, unsafe. Although preemptive extracorporeal support is often considered in situations of extreme airway narrowing, it introduces meaningful risks, including complications from vascular cannulation, bleeding, infection, and renal strain, and it may inadvertently encourage overly aggressive airway manipulation.

In contrast, this case demonstrates that a structured, hybrid awake strategy prioritizing preserved spontaneous ventilation, meticulous topical anesthesia, VV-ECMO standby rather than preemptive initiation, and low-profile intubation devices can achieve safe airway control without the morbidity associated with early ECMO cannulation. The combined use of video laryngoscopy, a microlaryngoscopy tube, and a Cook airway exchange catheter provided an atraumatic and adaptable alternative when a traditional adult bronchoscope could not be advanced. Given the rarity of tracheal chondrosarcoma and the high stakes of managing near-obstructing central airway disease, this case supports a selective, risk-stratified approach to ECMO utilization and contributes meaningful guidance for future airway management in similarly complex pathology.
